# What Protective Health Measures Are Americans Taking in Response to COVID-19? Results from the COVID Impact Survey

**DOI:** 10.3390/ijerph17176295

**Published:** 2020-08-29

**Authors:** Fares Qeadan, Nana Akofua Mensah, Benjamin Tingey, Rona Bern, Tracy Rees, Sharon Talboys, Tejinder Pal Singh, Steven Lacey, Kimberley Shoaf

**Affiliations:** Department of Family and Preventive Medicine, University of Utah, Salt Lake City, UT 84108, USA; nana.mensah@utah.edu (N.A.M.); Benjamin.Tingey@utah.edu (B.T.); rbzmb@mail.missouri.edu (R.B.); tracy.rees@utah.edu (T.R.); Sharon.Talboys@utah.edu (S.T.); tp.singh@utah.edu (T.P.S.); steven.lacey@utah.edu (S.L.); kimberley.shoaf@utah.edu (K.S.)

**Keywords:** protective health measures, SARS-CoV-2, COVID-19, public health, risk behavior

## Abstract

With the emergence of the novel SARS-CoV-2 and the disease it causes; COVID-19, compliance with/adherence to protective measures is needed. Information is needed on which measures are, or are not, being undertaken. Data collected from the COVID Impact Survey, conducted by the non-partisan and objective research organization NORC at the University of Chicago on April, May, and June of 2020, were analyzed through weighted Quasi-Poisson regression modeling to determine the association of demographics, socioeconomics, and health conditions with protective health measures taken at the individual level in response to COVID-19. The three surveys included data from 18 regional areas including 10 states (CA, CO, FL, LA, MN, MO, MT, NY, OR, and TX) and 8 Metropolitan Statistical Areas (Atlanta, GA; Baltimore, MD; Birmingham, AL; Chicago, IL; Cleveland and Columbus, OH; Phoenix, AZ; and Pittsburgh, PA). Individuals with higher incomes, insurance, higher education levels, large household size, age 60+, females, minorities, those who have asthma, have hypertension, overweight or obese, and those who suffer from mental health issues during the pandemic were significantly more likely to report taking precautionary protective measures relative to their counterparts. Protective measures for the three subgroups with a known relationship to COVID-19 (positive for COVID-19, knowing an individual with COVID-19, and knowing someone who had died from COVID-19) were strongly associated with the protective health measures of washing hands, avoiding public places, and canceling social engagements. This study provides first baseline data on the response to the national COVID-19 pandemic at the individual level in the US. The found heterogeneity in the response to this pandemic by different variables can inform future research and interventions to reduce exposure to the novel SARS-CoV-2 virus.

## 1. Introduction

In December 2019, a novel coronavirus known as severe acute respiratory syndrome coronavirus 2 (SARS-CoV-2) [1], emerged from Wuhan, China [2], crossing international borders, stalling economies worldwide, and infecting, as of 30 May 2020, over 6 million people worldwide and leading to COVID-19. As a respiratory illness, COVID-19 is chiefly spread through human-to-human contact via respiratory droplets, especially in indoor settings [3,4], as well as contact routes (e.g., touching of contaminated surfaces or objects) [5,6,7,8,9]. COVID-19 is a serious illness that causes a range of symptoms with the most commonly reported including fever, cough, fatigue, shortness of breath, and headaches. Gastrointestinal effects such as diarrhea and vomiting have also been reported [10], as have loss of smell or taste [11,12]. Reports from China and emerging data in the United States suggest that older adults and individuals with underlying health conditions are at a higher risk for severe COVID-19 related illnesses compared to younger individuals [13,14,15]. In the U.S., 8 out of 10 deaths were in those 65 years of age or older and those who are obese or have chronic kidney disease, obstructive pulmonary disease, compromised immune systems, heart conditions, or type 2 diabetes are at increased risk of having a severe case of COVID-19 regardless of age [13,15]. Given that older age and such a wide range of underlying health conditions increase the risk for or severity of COVID-19, the current pandemic poses a life-threatening danger to multiple vulnerable subpopulations within the United States.

Without a currently evidence-based and proven vaccine or pharmacological treatment, the main approach for stopping the spread of COVID-19 has been through behavioral changes [16], whether they be voluntary or involuntary. For example, the World Health Organization (WHO) and Centers for Disease Control and Prevention (CDC) have issued guidelines focused on the behavior of businesses, organizations, and individuals with an especially urgent need to protect vulnerable populations (i.e., older people and those with certain underlying health conditions) [17,18]. WHO guidelines underscore the importance of personal protective equipment (PPE) by healthcare workers and the general public [19], frequent hand hygiene, respiratory etiquette, environmental cleaning and sanitization, and maintaining physical distancing protocols [18]. Similarly, the CDC has urged people to stay at home when ill [17] and use cloth masks in communal and social settings where the recommended 6 feet of physical distance is difficult or impossible to maintain [20].

In March, the president and all 50 governors declared a state of emergency [21], which is defined as any event for which the president deems it necessary to provide additional federal assistance to protect property and/or public health and safety in an effort to lessen or avert catastrophe [22]. Although the U.S. developed pandemic plans in response to the 2003 severe acute respiratory syndrome (SARS) and the growing avian influenza threat, preparedness for and response to viral disasters [23] varies greatly from country to country and even state to state [24,25] here in the U.S. As of 1 April 2020, 33 states had issued statewide stay-at-home orders and 13 states had issued partial stay-at-home orders [26]. The pandemic disaster response has taken the form of voluntary international, national, and local guidelines and some involuntary policies, regulations, and orders (e.g., wearing facemasks, working from home, and closing of non-essential businesses) [27,28,29,30]. At the macrolevel, that is, at the federal, state, and local government level, the official disaster response has led much of the public to stay at home, the closing of schools, universities, and businesses, and changed operating procedures for businesses and organizations (e.g., limiting the number of patrons, moving to carryout food services, increasing and regularly sanitizing common areas, and requiring masks for employees and customers), as well as the cancelation of mass gatherings [21]. Despite these voluntary and involuntary protective health measures, by 3 June the United States had recorded a total of 1,831,821 cases and 106,181 deaths—the highest number of positive cases and deaths reported in any country [31]. 

What is less clear is what is happening at the microlevel, that is, to what extent individuals have been conforming with stay at home orders and other protective health measures. Thus far, very limited research has reported on the measures individuals are taking in response to COVID-19 [32,33]. This, however, is a critical question and with a positive case count and death toll that seems to increase daily, we undertook the task of examining the behavioral patterns of individuals in response to the pandemic. We had two objectives; first, we evaluated the relationship between the count of protective health measures taken at the individual level in response to COVID-19 and predictors of interest such as a COVID-19 diagnosis or proximity to someone with a diagnosis and other relevant demographic and clinical characteristics. The selection of these predictors was based on available data. Here, we hypothesize that individuals who have been infected with COVID-19 or know someone with or who had COVID-19 will likely take additional and more stringent protective health measures compared to those who have not been diagnosed or not known someone else with the disease. Second, we employed a network model to determine the interactions between each of the COVID-19 protective health measures, as when dealing with individual behaviors that are inherently relational, network analyses are particularly useful [34]. We further apply the network model (i.e., analysis of single protective health measures to create a network of interrelated measures) to subgroups comprised of (i) individuals with a COVID-19 diagnosis, (ii) individuals living with someone with a COVID-19 diagnosis, and (iii) individuals with a family member or close friend who died of COVID-19 to assess differences and similarities across groups. Research has shown that such individual-level analyses can uncover pivotal insights that would otherwise have been obscured by other analytic approaches [35,36]. Thus, a network model of protective health measures taken in response to COVID-19 can reveal critical patterns among measures such as which measures are most closely related to each other and which protective health measures are predictive of other measures.

## 2. Materials and Methods

### 2.1. Settings

We used data from the Data Foundation’s national COVID Impact Survey [37], which provides COVID-19 related data, along with data on physical health, mental health, economic security, and social dynamics. Data were collected from individual respondents’ answers to various survey questions, but different weights were used to reflect data on the national or regional levels. Data collection occurred in three ensuing waves (months of April, May, and June) to present a snapshot of the global pandemic’s impact on the U.S. populace’s experience of COVID-19 and how its effects on physical and mental health, economic and food security, and employment during each wave. The three waves are a series if independent cross sectional studies such that an overlap between them is less likely while possible. For each wave, data collection occurred over a week long period where interviews were conducted in English and Spanish. Respondents were offered a small monetary incentive for completing the survey while noting that data were collected using the AmeriSpeak Panel, which is a nationally representative and probability-based survey panel. Initially, randomly selected U.S. households were sampled from the non-partisan and objective research organization NORC at the University of Chicago National Sample Frame and then contacted by U.S. mail, email, telephone, and field interviewers (face-to-face). People with P.O. Box only addresses, addresses not listed in the USPS Delivery Sequence File, and some newly constructed dwellings were excluded from the sample. In households with more than one adult panel member, only one was selected at random for the sample. All invited members had the chance to complete the survey online or by telephone with an NORC telephone interviewer. An iterative raking process (utilizing age, gender, census division, race/ethnicity, education, and county groupings) was used to adjust for any survey nonresponse and any noncoverage or under and oversampling resulting from the study specific sample design [38]. The survey is designed to provide weekly estimates of the U.S. adult household population nationwide and for 18 regional areas including 10 states (CA, CO, FL, LA, MN, MO, MT, NY, OR, and TX) and Metropolitan Statistical Areas (Atlanta, GA; Baltimore, MD; Birmingham, AL; Chicago, IL; Cleveland and Columbus, OH; Phoenix, AZ; and Pittsburgh, PA). Our study employed a series of three cross-sectional studies on various protective health measures people are taking during the COVID-19 pandemic, and the impact various demographic, clinical, and socioeconomic characteristics have on those protective health measures.

### 2.2. Measurements

The outcome of interest was COVID-19 related protective measures being followed by individuals. This variable was based on the question “Which of the following measures, if any, are you taking in response to the coronavirus?” Respondents were able to select from 19 possible protective health measures including “cancelled a doctor’s appointment”, “wore a face mask”, “canceled or postponed school activities”, “worked from home”, “washed or sanitized hands”, “kept six feet distance from those outside my household”, and “avoided contact with high-risk people”. For the first analysis, the primary outcome of interest was the count of COVID-19 protective measures being taken. Thus, the 19 protective health measures indicated with a “yes” were summed for each respondent to reflect the count of all measures being taken by that individual. For the second analysis, the measures were left in their original survey form.

Predictors of interest included age, gender, race and ethnicity, median household income, education, household size, population density, regional location, insurance status, health conditions, and COVID-19 related information such as diagnosis or proximity to someone with a diagnosis. Other predictors were temporary life changes during COVID-19, and these variables were constructed similarly to the outcome of interest by summing or averaging indications across each respondent (Table A1).

All of the variables in their original survey form were categorical, but variables that were constructed (the outcome and the temporary life changes during COVID-19) were transformed into continuous ordinal variables. Household income and household size were also converted into continuous ordinal variables.

### 2.3. Statistical Analysis

The first analysis was done with all three waves of data. Overall demographics and clinical characteristics of participants for all three waves were calculated as an aggregate. Categorical variables were presented with frequencies of the raw respondent counts and weighted percentages to reflect the percentages of the regional adult population sizes. The means and standard deviations for continuous variables were also calculated.

To provide estimates of a variable’s impact on the count of COVID-19 protective health measures taken, a Quasi-Poisson regression model was fit and weighted to reflect the regional adult population. The analysis was stratified by time, and was run for each month to show how the estimates changed over time. Variables were selected by a forward selection method with a Schwarz–Bayesian information criterion, as well as by clinical relevance. Adjusted incidence rate ratios (aIRR) with 95% confidence intervals (CI) and *p*-values were calculated. Model fit and diagnostics were assessed.

The second analysis was also performed with all three waves of data, but included all the data aggregated together. To assess the structure of the COVID-19 protective health measures in their original form (rather than as an aggregate), a network analysis [39] was performed to determine the relationship between each of the COVID-19 measures. This was done across all of the data, as well as by subsets (i.e., (i) those with a COVID-19 diagnosis, (ii) those living with someone with a COVID-19 diagnosis, (iii) and those with a family member or close friend that died due to COVID-19). The network (or structure) of the COVID-19 measures were compared and contrasted between each of these different groups to see how the relationships were similar or changed. In each network, the nodes represented each of the COVID-19 protective health measures and the edges represented the pairwise relationships between measures. The networks were built using a regularized Ising model [40] (p. 617) that calculated partial correlations between each node, while adjusting for all other measures. Small relationships were penalized (by being sent to 0) so as to provide a robust indication of the real relationships amongst data points, thereby avoiding spurious relationships.

To identify the impact of each protective health measure in the network, standardized centrality measures were calculated to represent how closely related each measure was in the entire network of all measures. Centrality was split into three different metrics, including strength, betweenness, and closeness. Strength represents the sum of all weighted edges, betweenness represents how short the path is from one measure to all other measures, and closeness represents how closely protective health measures are to each other on average [41] (pp. 245–251). Generally, protective health measures with higher indications of centrality are more predictive of all the other measures in the network.

For the count modeling, all hypothesis tests were two-sided with a significance level of 5% and SAS version 9.4 (SAS Institute, Inc., Cary, NC, USA) was used. The network analysis was performed in R, version 3.6.1 (R Foundation for Statistical Computing, Vienna, Austria).

## 3. Results

Of a total of 25,269 respondents, 29.0% (9942) of the regional adult population were 60 years of age or older, 51.3% (14,186) were female, 49.8% (15,985) were non-Hispanic white, and 11.4% (2290) were non-Hispanic black. There were 16.1% (4526) making between $50,000 and $75,000, and 31.3% (13,254) with a bachelor’s degree or above. The study population was primarily urban (82.9% (19,829)) and a major portion was from the southern region (38.4% (8161)). The majority had insurance (86.7% (23,464)) (Table 1).

**Figure 1:**
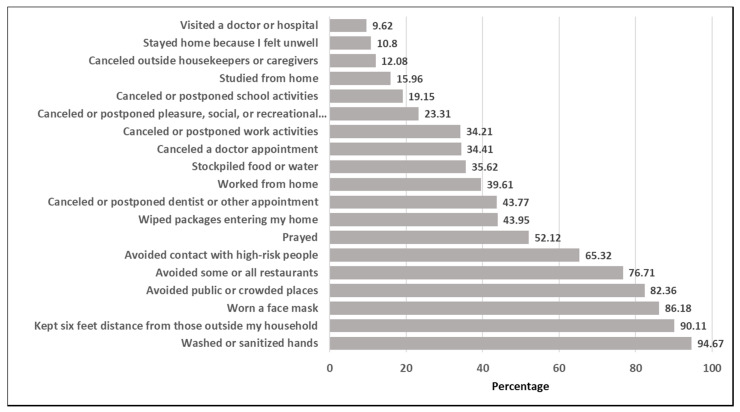
The proportions of individuals who reported positively to the 19 protective health measures taken in response to COVID-19.

As far as health indicators, 38.8% (10,443) reported very good health. The majority of respondents did not report any chronic conditions, although 29.5% (8434) had high blood pressure or hypertension, 13.6% (3429) had asthma, and 15.1% (4062) had a mental health condition. A large percentage was overweight or obese (30.1% (8250)). Smaller amounts of respondents had chronic lung disease or chronic obstructive pulmonary disease (COPD) (4.1% (1036)), bronchitis or emphysema (10.4% (2905)), and a compromised immune system (6.6% (1858)). There was a small percentage of those with a COVID-19 diagnosis (0.84% (181)) living with someone with COVID-19 (0.98% (175)), or who had a family member or close friend die from COVID-19 or a respiratory illness since March 1, 2020 (5.3% (1121)). A larger portion of respondents had plans that changed due to COVID-19 (mean count (SD): 6.9 (4.4)), whereas less were seeking financial aid (mean count (SD): 1.1 (1.5)) or experiencing flu-like symptoms (mean count (SD): 2.1 (2.5); Table 2). The proportions of individuals who reported positively to the 19 protective health measures taken in response to COVID-19 are displayed in Figure 1. About 94.7% of participants reported washing or sanitizing hands, 39.6% reported working from home, and 9.6% reported visiting a doctor or hospital. Summing the positive indications across the 19 measures for each individual revealed a total sample median of 9 measures taken with an interquartile range (IQR) of 4.

**Figure 2:**
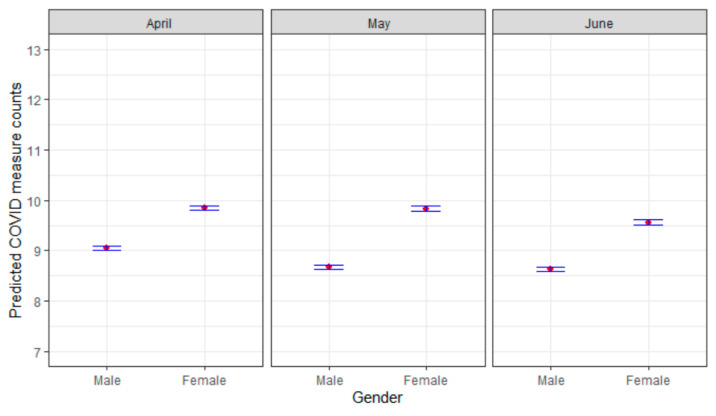

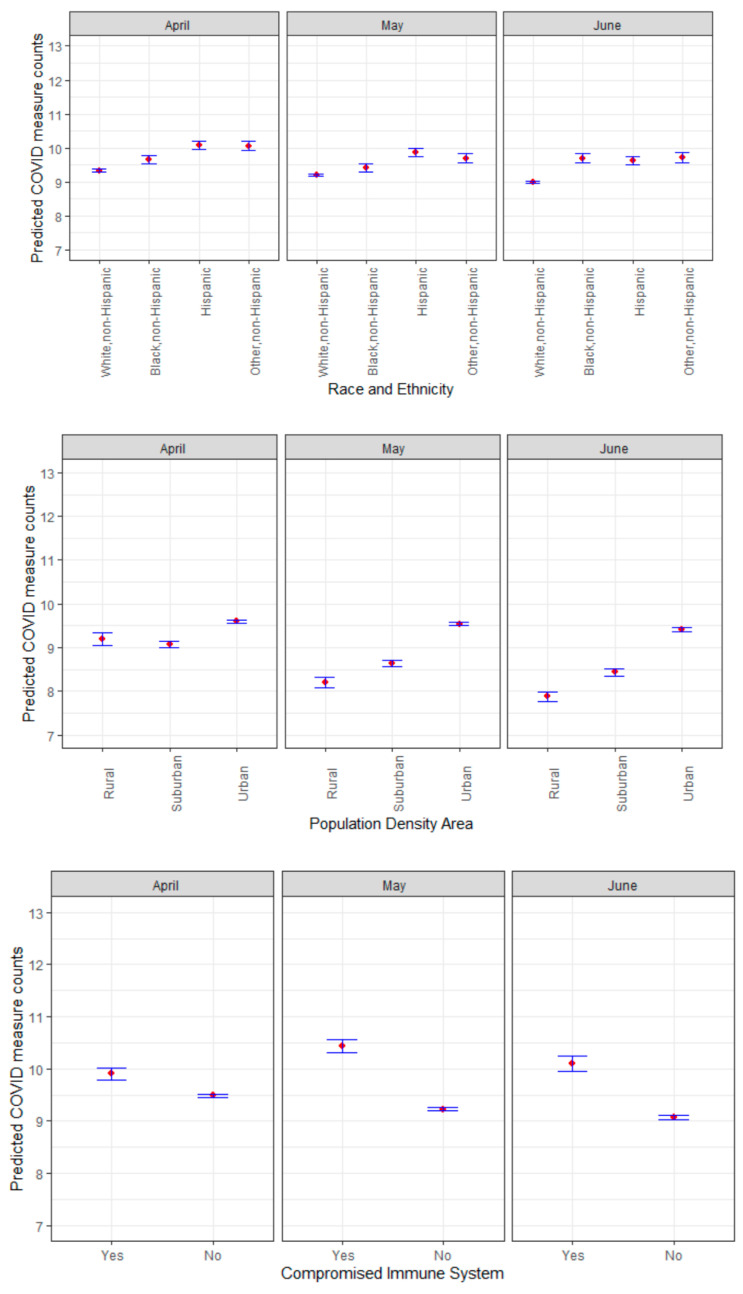

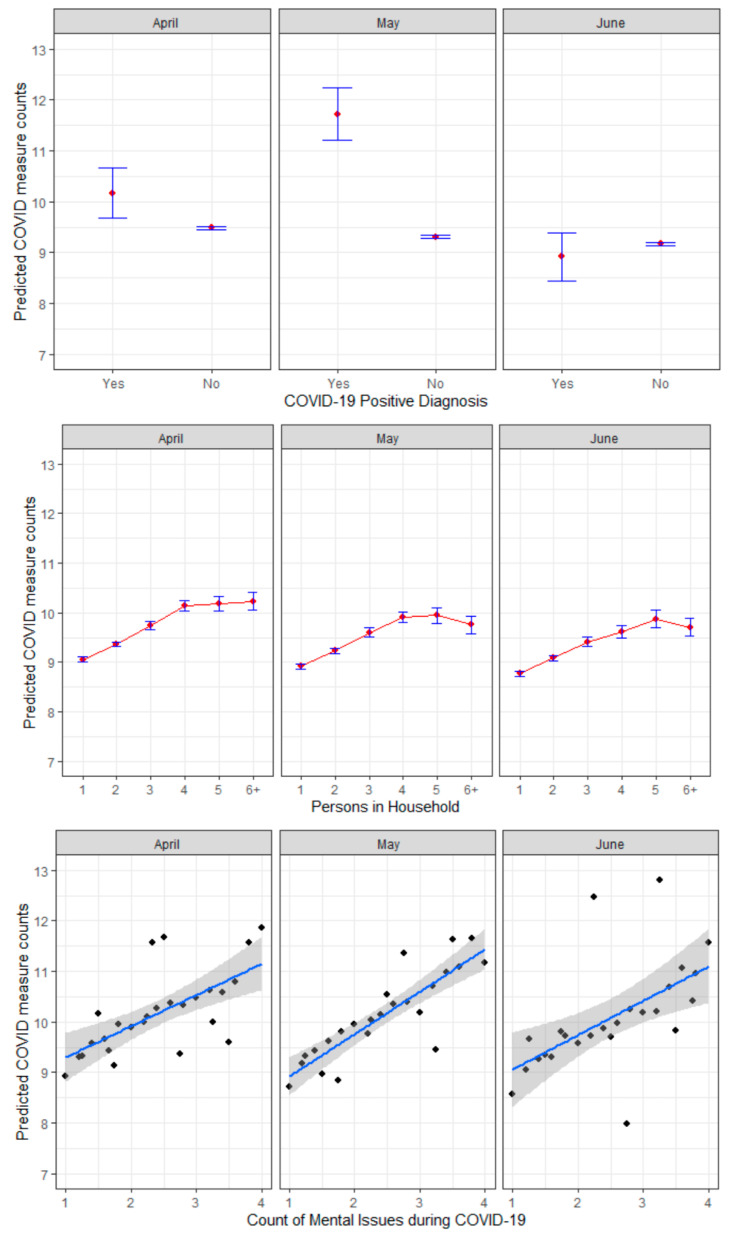
Variables’ associations with predicted COVID-19 protective measures count (with 95% confidence intervals).

Table 3 shows the adjusted IRRs of variables’ impact on the expected count of COVID-19 protective health measures taken over time (April, May, and June). By age, 30–44 year olds and 60 year olds and older took more protective health measures than 18 to 29 year olds (30–44 aIRRs: 1.03 (April), 1.05 (May), and 1.03 (June); 60+ aIRRs: 1.05 (April), 1.03 (May), and 1.04 (June)). Females took more protective health measures than males (aIRRs: 1.08 (April), 1.12 (May), 1.09 (June)). Blacks, Hispanics, and other non-Hispanic races took more protective health measures than whites, as well as those with higher levels of education taking more measures than those with no high school diploma. Those with some form of insurance had higher protective-health measure counts than those without (aIRRs: 1.15 (April), 1.08 (May), and 1.09 (June)). The bigger the household size and the greater the household income, the greater the protective health measures that were taken (household size aIRRs: 1.02 (April), 1.01 (May), and 1.01 (June); household income aIRRs: 1.01 (April), 1.02 (May), and 1.02 (June)). Rural and suburban residents had lower rates of protective health measures being taken than urban residents, especially after April (rural aIRRs: 1.02 (April), 0.92 (May), and 0.91 (June); suburban aIRRs: 0.98 (April), 0.95 (May), and 0.94 (June)). When comparing to the south, those living in the northeast/midwest/west generally had lower rates of protective health measures being taken. Those with high blood pressure/hypertension, asthma, a compromised immune system, and who were overweight or obese all saw consistently higher rates over time of protective health measures being taken than those without. Those who were diagnosed with COVID-19 had higher rates of protective health measures being taken in May (aIRR: 1.18, 95% CI [1.09, 1.28]) but lower rates in June (aIRR: 0.89, 95% CI [0.81, 0.97]). Those who had a close friend or family member die from COVID-19 also had higher rates of protective health measures across time (aIRRs: 1.04 (April), 1.02 (May), and 1.16 (June)). Increased counts of plans being changed and mental health issues saw increased rates of COVID-19 protective health measures being taken. Visual illustrations for the association between some of the variables in Table 3 and the predicted count of the primary outcome are provided in Figure 2.

**Figure 3:**
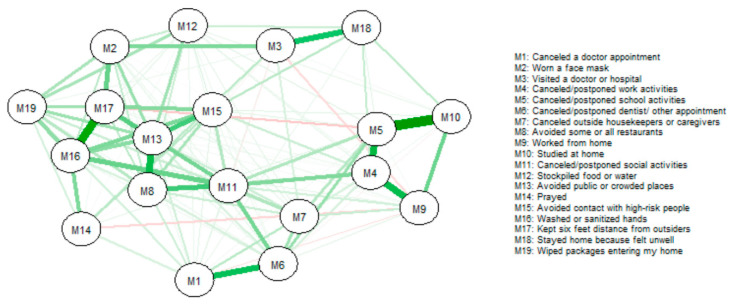
Network of COVID-19 measures taken for all survey respondents (*n* = 25,269). Line thickness represents the strength of pairwise COVID-19 measure connections (thicker = stronger, thinner = weaker), more green represents more positive pairwise connections; more red represents more negative pairwise connections.

**Figure 4:**
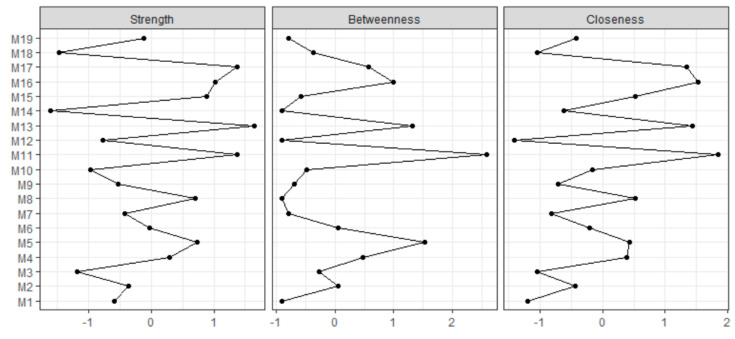
Standardized centrality for each COVID-19 measure taken for all respondents. **Centrality**: how connected each COVID-19 protective health measure is to other measures in the network (COVID-19 measures with higher centrality across the three centrality metrics are generally the best in predicting other COVID-19 protective health measures); strength centrality: the sum of edge weights of edges connecting to other COVID-19 protective health measures; betweenness centrality: how short edge paths are connecting one COVID-19 protective health measure to other measures; and closeness centrality: how close a COVID-19 protective health measure is to other measures on average.

The network of COVID-19 protective health measures undertaken by survey respondents across all time points is displayed in Figure 3. The strongest relationships included “washing/sanitizing hands” and “kept six feet distance from those outside the home”, as well as “cancelled/postponed school activities” and “studied from home”. Thus, washing/sanitizing hands is predictive of keeping distance from those outside the home (and vice versa) as well as cancelling school activities and studying from home (vice versa) across all respondents and adjusting for all other relationships. Indications of cancelling appointments or activities were predictive of each other and indications of avoiding crowds or groups were also predictive of each other. However, “cancelled/postponed school activities” was negatively and weakly related to “avoided contact with high-risk people”. The measures with the strongest centrality (across all three metrics) were “cancelled social activities”, “avoided public or crowded places”, and ”washed or sanitized hands”. Therefore, just these measures alone are likely to provide the information needed to predict other COVID-19 protective health measures taken (Figure 4).

**Figure 5:**
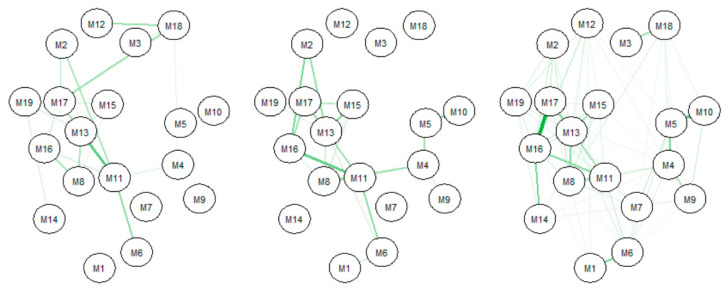
Network of COVID-19 measures taken by COVID-19 groups. (**Left**): COVID-19 diagnosis; (**Middle**): Live with COVID-19 diagnosis; and (**Right**): Family member/close friend die from COVID-19.

Given that participants who were either positive for COVID-19, know an individual with COVID-19, or know someone who had died from COVID-19 were more likely to adapt a stricter lifestyle than those who were not directly hit by the pandemic, it was important to learn about the network of COVID-19 protective health measures undertaken by such groups. Figure 5 shows the networks for (i) those with COVID-19, (ii) those living with someone with COVID-19, and (iii) those having a family member or close friend die from COVID-19. Of those with COVID-19, a relationship of note was the relationship between “stockpiling food and water” and “staying home because feeling unwell”. This relationship was not seen in the other groups. The strongest relationship, however, was between “cancelling or postponing social activities” and “avoiding public or crowded places”. Of those living with someone who has COVID-19, there was a relationship between “wearing a face mask” and “washing or sanitizing hands”. In the group having a family member or close friend die from COVID-19, the strongest relationship was “washing hands” and “keeping six feet distance”. There were relationships seen in all of the groups that related to avoiding group gatherings (i.e., cancelled/postponed social activities and avoided public or crowded places, avoided public or crowded places, and avoided restaurants).

**Figure 6:**
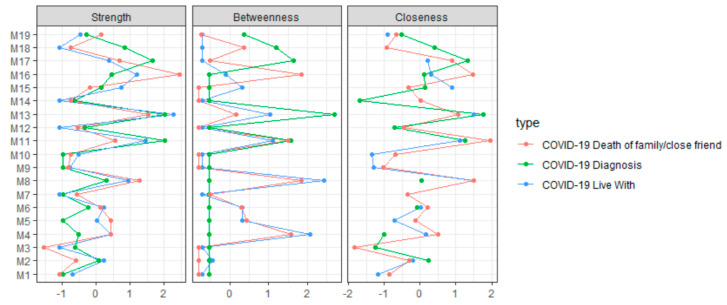
Standardized centrality for each COVID-19 measure taken by COVID-19 groups.

Figure 6, “avoided public or crowded places” tended to be strong across all groups. “Avoided some or all restaurants”, “canceled/postponed social activities”, and “washed or sanitized hands” were also strong across all groups.

## 4. Discussion

As SARS-CoV-2 is a recently identified beta-coronavirus in the same family as SARS-CoV and MERS-CoV, having emerged from China only in December 2019, studies on the behavioral responses of individuals have been limited. Although government agencies and local public health departments have recommended guidelines for reducing exposure or transmission, it is unclear to what extent individuals are incorporating recommended public health guidelines and the effect of the pandemic on individuals’ behavior. Thus, the present study addressed that gap and found significantly higher rates of public health measures being implemented (COVID-19 measures) were associated with demographic, socioeconomic, and clinical variables. The results of the protective health measures count analysis show varying demographic and socioeconomic variables associated with significantly higher rates of COVID-19 measures being taken. Older ages, females, Blacks/Hispanics/non-Hispanics all took a significantly higher number of protective measures in comparison to Whites and those with higher income, more education, bigger household sizes, and insurance. Barr et al. also cited younger adults as less likely to comply with protective health measures while individuals with higher education (university) were associated with a higher compliance [42]. Face mask compliance in an Australian study conducted after the SARs outbreak in Hong Kong and Canada was found to be especially high [42]. Geographic variables, however, showed associations with significantly lower rates of protective health measures, such as those living in suburban and rural communities when compared to urban areas, and all other regions when compared to the southern United States. Generally, these associations were robust over time with the exception of some regions and rural communities having positive rates in April. This could be correlated to an increase in coronavirus infections in the U.S. in April [43], which was the month of the first peak in some states [25]. As reported by the CDC, there was a 14 fold increase in the number of coronavirus infections, moving from 68,440 confirmed cases in March to 957,875 confirmed cases by the end of April (5 April 2020 case numbers 330,891; April 26 cases 957,875) [43].

The impact of a COVID-19 diagnosis had variable results over time. Those with a positive COVID-19 diagnosis had much higher rates of measures taken in May, but the opposite was true in June, which saw much lower rates. The impact of clinical factors on protective health measures being taken was seen in those with pre-existing conditions such as asthma, overweight/obesity, hypertension, and a compromised immune system all showing higher rates across the three survey periods. The greater amount of plans that had to be changed and indications of mental health stressors also showed higher rates across time. The network analysis showed many intuitive relationships: cancelling work/school and working/studying from home, washing/sanitizing hands and keeping 6-feet distance from people. When separating between COVID-19 classes, those with a COVID-19 diagnosis had stronger emphases on self-isolation (cancelling social events, avoiding large crowds, staying home, and stockpiling food and water), whereas those living with someone with COVID-19 or having a close friend or family member dying from COVID-19 had stronger emphases on personal protective health measures (washing/sanitizing hands, wearing a face mask, and six-feet distancing). These results conform to those of a 2008 study on community response to a hypothesized influenza pandemic conducted nationwide in which Blendon et al. found that 94% of respondents would self-isolate if infected and 85% would remain at home with a sick household member [44]. Common to all three groups and the strongest were “cancelled social activities”, “avoided public or crowded places”, and “hand sanitizing”, which is suggestive of predicting other measures taken in response to the coronavirus. Our mental health findings also support prior research: A study conducted in Hong Kong during the 2009 H1/N1 outbreak found a strong and consistent association with mental health indicators (“anticipated worry”, “experienced worry”, and “current worry”) in the adoption of protective health measures including sanitizing and avoiding crowds [45].

If individuals are to respond appropriately in the mitigating of virus transmission, the implementation of public health measures needs to be considered with the factors described (i.e., proper hygiene with hand washing, avoiding social gatherings, and isolating when infected). In addition, previous studies have been published on individual knowledge and behavior in relation to SARs and Avian influenza [42,46]. More recent studies have also specifically focused on behavior responses of the public and attitude to the 2009 swine influenza pandemic [45,47,48,49,50]. Hypothetical pandemic scenarios have been studied as well [44,51]. Their work shows that with a poor behavioral response to public health measures, the number of cases will increase.

Our findings and those of others suggest that demographics and socioeconomics influence public behavior and protective health measures taken by and within the community. Future studies are needed to better determine compliance with public health measures to limit the effects of pandemics. With the outbreak of the 1997 avian influenza (H5N1), the 2003 SARS coronavirus, MERS (2012), and Swine influenza (2009), it has been demonstrated that communication by public health officials and protective health measures taken at the individual level can limit the effects of a pandemic.

This study has some limitations. The survey was conducted via the internet and telephone interviews in selected states, which could result in selection bias. In addition, the survey did not include all 50 states or U.S. territories. In addition, the data were self-reported making it difficult to be independently verified and is subject to recall bias. This study used secondary data analysis and is constrained by the available variables set out in the survey. As the survey was conducted over a 3-month period but used a different sample of the population for each period, longitudinal effects cannot be established. As the COVID-19 pandemic is a rapidly evolving situation with national and local policies being constantly modified, the surveys used may not fully capture the current situation. Currently, there is a lack of research studies on public health measures taken in response to COVID-19. Larger population-based studies that include areas where individuals are not able to access the internet could clarify regional area differences in public health measures taken by communities. Follow-up studies are needed to assess a rapidly evolving and dynamic situation.

## 5. Conclusions

Data from this study provide the first baseline on response to a national pandemic at the individual level. These data shed light on and can be used for measuring and increasing compliance in public health measures that mitigate virus transmission. There may be a need for frequent surveys with a rapid turnaround for a timely gauge of the community’s response in adopting protective health measures and to determine the need for widespread communication efforts. This pandemic is a rapidly evolving situation and future research will need to be performed quickly if optimal adherence to public health measures that mitigate transmission and protect community health are to be followed at the community and individual level.

## Figures and Tables

**Table 1:** Demographics of participants.

Total	25,269 ^1^ (100.00%)
**Age (years)**	
18 to 29	3226 ^1^ (20.79) ^2^
30–44	6117 (27.13)
45–59	5981 (23.06)
60+	9942 (29.01)
**Gender**	
Male	11,070 (48.73)
Female	14,186 (51.27)
**Race and Ethnicity**	
Non-Hispanic White	15,985 (49.77)
Non-Hispanic Black	2290 (11.42)
Hispanic	2258 (23.03)
Non-Hispanic Other	1789 (9.69)
Unknown ^3^	2947 (6.10)
**Household Income**	
Under $10,000	1283 (8.63)
$10,000 to under $20,000	1809 (9.54)
$20,000 to under $30,000	2360 (12.03)
$30,000 to under $40,000	2240 (9.50)
$40,000 to under $50,000	1942 (8.08)
$50,000 to under $75,000	4526 (16.09)
$75,000 to under $100,000	3568 (12.19)
$100,000 to under $150,000	3866 (12.13)
$150,000 or more	3055 (9.75)
Unknown	620 (2.06)
**Education**	
No high school diploma	885 (9.83)
High school graduate or equivalent	3263 (28.65)
Some college	7828 (30.26)
BA or above	13,254 (31.26)
**Household Size**	
One person (I live by myself)	7711 (28.15)
Two persons	8860 (30.10)
Three persons	3514 (15.82)
Four persons	2638 (12.10)
Five persons	1295 (7.00)
Six or more persons	1203 (6.83)
**Population Density**	
Rural	1445 (4.15)
Suburban	3990 (12.96)
Urban	19,829 (82.88)
**Region**	
Northeast	3055 (13.52)
Midwest	7036 (14.71)
South	8161 (38.36)
West	7017 (33.40)
**Insurance**	
No	1805 (13.31)
Yes	23,464 (86.69)

^1^ Raw survey sample size (combined from April, May, and June), counts may not add up to total study sample size due to removal of missing values; ^2^ column %’s (weighted to regional adult population); ^3^ non-response.

**Table 2:** Clinical and other characteristics of participants.

Total	25,269 ^1^
**Overall Self-Reported Health**	
Excellent	4992 ^1^ (20.25) ^2^
Very good	10,443 (38.80)
Good	6880 (28.34)
Fair	2404 (10.01)
Poor	522 (2.60)
**COVID-19 positive**	
Yes	181 (0.84)
No	24,899 (98.04)
Unknown ^3^	189 (1.12)
**COVID-19 positive of someone living with**	
Yes	175 (0.98)
No	24,714 (97.22)
Unknown	380 (1.79)
**COVID-19 or respiratory illness death of family member or close friend since 1 March 2020**	
Yes	1121 (5.27)
No	23,621 (92.13)
Unknown	527 (2.60)
**Plans having been changed due to COVID-19 ^4^ mean (SD)**	6.89 (4.42)
**Sought financial aid during COVID-19 ^4^ mean (SD)**	1.07 (1.49)
**Flu-like symptoms during COVID-19 ^4^ mean (SD)**	2.12 (2.45)
**Interest in COVID-19 management measures ^5^ mean (SD)**	2.98 (1.14)
**Mental health issues during COVID-19 ^5^ mean (SD)**	1.51 (0.64)
**Diabetic**	
Yes	2803 (11.15)
No	21,769 (85.43)
Unknown	697 (3.41)
**High blood pressure or Hypertension**	
Yes	8434 (29.53)
No	16,114 (66.59)
Unknown	721 (3.89)
**Heart disease, heart attack, or stroke**	
Yes	2036 (6.77)
No	22,435 (89.38)
Unknown	798 (3.85)
**Asthma**	
Yes	3429 (13.61)
No	21,078 (82.54)
Unknown	762 (3.85)
**Chronic lung disease or COPD**	
Yes	1036 (4.09)
No	23,631 (93.04)
Unknown	602 (2.87)
**Bronchitis or emphysema**	
Yes	2905 (10.41)
No	21,777 (86.69)
Unknown	587 (2.89)
**Allergies**	
Yes	11,227 (41.42)
No	13,327 (55.21)
Unknown	715 (3.37)
**Mental health condition**	
Yes	4062 (15.09)
No	20,460 (80.92)
Unknown	747 (3.99)
**Cystic fibrosis**	
Yes	74 (0.50)
No	24,807 (97.24)
Unknown	388 (2.25)
**Liver disease or end stage liver disease**	
Yes	327 (1.39)
No	24,559 (96.62)
Unknown	383 (1.99)
**Cancer**	
Yes	2344 (6.40)
No	22,443 (91.14)
Unknown	482 (2.46)
**Compromised immune system**	
Yes	1858 (6.62)
No	22,713 (89.97)
Unknown	698 (3.41)
**Overweight or obese**	
Yes	8250 (30.12)
No	16,469 (66.91)
Unknown	550 (2.97)

^1^ Raw survey sample size (combined from April, May, and June), counts may not add up to total study sample size due to removal of missing values; ^2^ column %’s (weighted to regional adult population); ^3^ non-response; ^4^ sum of indications; ^5^ average of indications.

**Table 3:** Adjusted estimates of variables’ impact on COVID-19 protective measures count stratified by month ^1^.

Variables	April Adjusted IRR ^2^ (95% CI)	May Adjusted IRR ^2^ (95% CI)	June Adjusted IRR ^2^ (95% CI)
**Age (years)**			
18 to 29	1 [Reference]	1 [Reference]	1 [Reference]
30–44	**1.03 (1.01, 1.05)**	**1.05 (1.03, 1.08)**	**1.03 (1.00 ** ^**3**^ **, 1.06)**
45–59	**1.04 (1.02, 1.07)**	1.00 (0.98, 1.03)	1.02 (0.99, 1.05)
60+	**1.05 (1.02, 1.08)**	**1.03 (1.00 ** ^**3**^ **, 1.06)**	**1.04 (1.01, 1.07)**
**Gender**			
Male	1 [Reference]	1 [Reference]	1 [Reference]
Female	**1.08 (1.06, 1.09)**	**1.12 (1.10, 1.14)**	**1.09 (1.07, 1.11)**
**Race and Ethnicity**			
Non-Hispanic White	1 [Reference]	1 [Reference]	1 [Reference]
Non-Hispanic Black	**1.03 (1.01, 1.06)**	1.00 (0.97, 1.03)	**1.05 (1.02, 1.08)**
Hispanic	**1.09 (1.07, 1.12)**	**1.06 (1.04, 1.08)**	**1.06 (1.03, 1.08)**
Non-Hispanic Other	**1.07 (1.04, 1.10)**	**1.04 (1.01, 1.07)**	**1.06 (1.03, 1.09)**
Household income	**1.01 (1.01, 1.01)**	**1.02 (1.01, 1.02)**	**1.02 (1.01, 1.02)**
**Education**			
No high school diploma	1 [Reference]	1 [Reference]	1 [Reference]
High school graduate or equivalent	**1.08 (1.04, 1.11)**	0.99 (0.96, 1.02)	0.98 (0.95, 1.02)
Some college	**1.10 (1.07, 1.14)**	**1.03 (1.00 ** ^**3**^ **, 1.07)**	1.02 (0.99, 1.06)
BA or above	**1.16 (1.12, 1.20)**	**1.11 (1.07, 1.14)**	**1.09 (1.05, 1.13)**
**Household size**	**1.02 (1.01, 1.03)**	**1.01 (1.00 ** ^**3**^ **, 1.01)**	**1.01 (1.01, 1.02)**
**Population density**			
Urban	1 [Reference]	1 [Reference]	1 [Reference]
Suburban	***0.98 (0.95, 1.00 ^3^)***	**0.95 (0.93, 0.98)**	**0.94 (0.92, 0.97)**
Rural	1.02 (0.97, 1.06)	**0.92 (0.88, 0.96)**	**0.91 (0.87, 0.96)**
**Region**			
South	1 [Reference]	1 [Reference]	1 [Reference]
Northeast	***1.02 (1.00 **^**3**^**, 1.05)***	0.99 (0.97, 1.02)	0.98 (0.95, 1.01)
Midwest	0.98 (0.95, 1.00 ^3^)	**0.97 (0.95, 1.00 **^**3**^)	0.99 (0.96, 1.02)
West	0.99 (0.97, 1.01)	**0.95 (0.93, 0.97)**	**0.95 (0.93, 0.97)**
**Insurance**			
No	1 [Reference]	1 [Reference]	1 [Reference]
Yes	**1.15 (1.12, 1.18)**	**1.08 (1.05, 1.11)**	**1.09 (1.06, 1.12)**
**High blood pressure or Hypertension**			
No	1 [Reference]	1 [Reference]	1 [Reference]
Yes	**1.04 (1.02, 1.06)**	**1.03 (1.01, 1.05)**	***1.02 (1.00 **^**3**^* **, ** ***1.04)***
**Asthma**			
No	1 [Reference]	1 [Reference]	1 [Reference]
Yes	**1.05 (1.03, 1.07)**	**1.05 (1.02, 1.07)**	**1.04 (1.02, 1.07)**
**Chronic lung disease or COPD**			
No	1 [Reference]	1 [Reference]	1 [Reference]
Yes	***1.04 (1.00 **^**3**^**, 1.08)***	1.01 (0.96, 1.06)	1.00 (0.96, 1.04)
**A mental health condition**			
No	1 [Reference]	1 [Reference]	1 [Reference]
Yes	1.00 (0.98, 1.03)	1.01 (0.98, 1.03)	1.01 (0.98, 1.04)
**A compromised immune system**			
No	1 [Reference]	1 [Reference]	1 [Reference]
Yes	1.03 (0.99, 1.06)	**1.10 (1.06, 1.13)**	**1.08 (1.04, 1.11)**
**Overweight or obese**			
No	1 [Reference]	1 [Reference]	1 [Reference]
Yes	**1.04 (1.02, 1.06)**	**1.05 (1.03, 1.07)**	**1.02 (1.00 ** ^**3**^ **, 1.04)**
**COVID-19 positive diagnosis**			
No	1 [Reference]	1 [Reference]	1 [Reference]
Yes	1.00 (0.91, 1.11)	**1.18 (1.09, 1.28)**	**0.89 (0.81, 0.97)**
**COVID-19/respiratory illness death of friend or close friend since March 1, 2020**			
No	1 [Reference]	1 [Reference]	1 [Reference]
Yes	**1.04 (1.00 ** ^**3**^ **, 1.08)**	1.02 (0.99, 1.06)	**1.16 (1.11, 1.20)**
**Plans having been changed due to COVID-19**	**1.02 (1.02, 1.03)**	**1.02 (1.02, 1.03)**	**1.02 (1.02, 1.02)**
**Mental health issues during COVID-19**	**1.07 (1.05, 1.08)**	**1.06 (1.04, 1.07)**	**1.07 (1.05, 1.08)**
**R ^2^**	**20%**	**19%**	**17%**

^1^ Quasi-Poisson regression model weighted by regional adult population, variables selected by forward selection method with Schwarz-Bayesian information criterion, as well as by clinical relevance; ^2^ incidence rate ratio, ^3^ marginally significant, or on the boundary of significance showing 1 due to rounding off: April *p*-values: suburban: 0.052, northeast: 0.059, midwest: 0.11, chronic obstructive pulmonary disease (COPD): 0.08, COVID-19 death of friend: 0.03; May *p*-values: age 60+: 0.02, some college: 0.046, household size: 0.02, midwest: 0.03; June *p*-values: age 30–44: 0.02, hypertension: 0.08, overweight or obese: 0.03. Note: Bold data indicate statistical significance; italic data indicate on the boundary of significance.

## Appendix A

**Table A1:** Construct variables information.

**Primary Outcome**
COVID-19 measure counts
Sum of “Yes” indications across M1–M19 relating to COVID-19 measures taken
Exposures
Insurance
“Yes” if any of PHYS9A–PHYS9H were “Yes, otherwise “No” (relating to insurance types respondents have)
Plans having been changed over past 7 days
Sum of “Yes” indications across ECON8A–ECON8S relating to instances in which plans have been changed
Sought financial aid over past 7 days
Sum of all indications across ECON6A–ECON6L relating to applying for aid or trying to apply for aid
Flu-like symptoms over past 7 days
Sum of “Yes” indications from PHYS1A–PHYS1Q relating to flu-like symptoms
Interest in COVID-19 management measures
Converted PHYS10A–PHYS10E into 5 point scale: “Extremely likely” to 5 and “Not likely at all” to 1, then averaged
Mental health issues over past 7 days
Converted SOC5A—SOC5E into 4 point scale: “5–7 days” to 4 and “not at all or less than 1 day” to 1, then averaged
